# Introducing transbronchial cryobiopsies in diagnosing diffuse parenchymal lung diseases in Greece: Implementing training into clinical practice

**DOI:** 10.1371/journal.pone.0217554

**Published:** 2019-06-03

**Authors:** Konstantinos Samitas, Lykourgos Kolilekas, Ioannis Vamvakaris, Charalampos Gkogkou, Petros Filippousis, Mina Gaga, Eleftherios Zervas

**Affiliations:** 1 Respiratory Medicine Dept. and Asthma Center, Athens Chest Hospital “Sotiria”, Athens, Greece; 2 Central Bronchoscopy Unit, Athens Chest Hospital “Sotiria”, Athens, Greece; 3 Pathology Dept., Athens Chest Hospital “Sotiria”, Athens, Greece; 4 Pathology Dept., HistoBioDiagnosis Laboratory, Athens, Greece; 5 Dept. of Medical Imaging and Interventional Radiology, Athens Chest Hospital “Sotiria”, Athens, Greece; University of British Columbia, CANADA

## Abstract

**Introduction:**

Diffuse parenchymal lung diseases (DPLD) constitute a heterogeneous group of disorders, sometimes requiring surgical lung biopsies (SLB) to obtain a definite diagnosis. Transbronchial cryobiopsy (TBCB) is a new promising interventional bronchoscopic method of obtaining lung tissue that is gaining ground against SLB.

**Methods:**

Fifty consecutive patients with indeterminate DPLD (definite/possible UIP excluded), after expert panel review referral, were retrospectively analyzed from January 2016 to August 2018. Patients underwent TBCB under deep sedation with endotracheal intubation and spontaneous breathing at a single, tertiary-care, reference hospital.

**Results:**

A total of 110 TBCBs (2.7 per patient, range 1 to 4) were performed. Frequent complications included mild pneumothorax in 5 patients (10%), requiring only oxygen supplementation, and bleeding in 31 patients (62%) that was mild in 19 patients and moderate in 12 patients. No serious bleeding was observed. There was zero mortality and no serious adverse events. Adequate samples for diagnostic purposes were obtained in 46 patients (92%) and pathologic histologic diagnosis was reached in 40 patients (80%). The most frequent histopathological patterns were organizing pneumonia (OP) (25%) and non-specific interstitial pneumonia (NSIP) (15%). After an expert panel review of all cases a final diagnosis was achieved in 38 patients, corresponding to a diagnostic yield of 76% for TBCB.

**Conclusion:**

Our single center cohort demonstrates that establishing TBCBs as a new technique is safe and feasible after proper training in specialized centers, resulting in low complication rates and adequate diagnostic yields.

## Introduction

Diffuse parenchymal lung diseases (DPLD) are a heterogeneous group of lung disorders, comprising more than two hundred different diseases. They are characterized by varying degrees of inflammation and fibrosis, primarily affecting the lung interstitium, although the alveolar space, bronchioles and pulmonary vessels can also be involved [1]. In everyday clinical practice, the differential diagnosis of DPLDs is complex and is based on the joint analysis of clinical, radiological and laboratory characteristics, usually in the context of a multidisciplinary team (MDT) discussion [1]. In certain cases, the definitive diagnosis of DPLD can be established only through histopathological examination of lung biopsy specimens. The options for lung biopsy until recently were limited to bronchoscopy and transbronchial biopsy (TBB) using forceps usually under fluoroscopy, and surgical lung biopsy (SLB), usually through video-assisted thoracoscopy. The latter is considered the ‘silver standard’ diagnostic approach in the differential diagnosis of DPLDs while the ‘gold standard’ is the multidisciplinary discussion.

Conventional TBBs with forceps are gradually being abandoned in the algorithm of DPLD diagnosis as their diagnostic yield in such peripheral and heterogeneous disorders is limited due to sample size and crush artifacts [2,3]. SLB has been the preferred method for histology for many years; however, it typically requires general anesthesia and hospitalization, resulting in increased costs [4]. Moreover, despite its high diagnostic yield [5], SLB is associated with major complications such as acute exacerbation of underlying fibrotic disease, persistent air leak, hemothorax, postoperative pneumonia and pneumothorax after discharge, and in many patients the risk/benefit ratio of the procedure is simply unacceptable [6]. Morbidity and mortality related to SLB are substantial, particularly in older subjects, in patients with significant comorbidities or severe respiratory impairment, and in cases with a final diagnosis of usual interstitial pneumonia/pulmonary fibrosis (UIP/PF) [7]. Essentially, SLB for fibrotic interstitial lung diseases (fILD) has a similar mortality to lobectomy for lung cancer, and clinicians and patients should understand the likely risks involved [8]. Therefore, less invasive procedures yielding comparable diagnostic information are needed, in a *primum non nocere* diagnostic approach.

The recent introduction of transbronchial cryobiopsies (TBCB) as a promising and safer alternative to SLB has generated considerable interest in the pulmonary community [9]. Samples retrieved by this method are significantly larger than by conventional TBB and usually without crush artifacts [10,11]. Complications such as bleeding and pneumothorax have been reported, but at a significantly lower rate when compared to SLB [12,13]. Therefore, although its diagnostic yield may lag behind that of SLB, TBCB could be considered as an alternative in the evaluation of patients with DPLD due to its acceptable safety and potential cost saving profile, probably in a ‘two-step’ approach with TBCB preceding SLB [12,14–17]. TBCB should be considered in a case-by-case basis, in order to avoid delays on definitive diagnosis and increased risk of complications due to this stepwise approach.

Despite its promising usefulness in DPLD diagnosis; however, the TBCB technique had not until recently been standardized and techniques, reported diagnostic yields, and complications vary widely [18]. A recently published Expert Statement was the first comprehensive attempt to put things into perspective regarding the role of TBCB in the diagnostic evaluation of DPLDs, proper patient selection, contraindications and safety considerations and finally how and who should perform TBCB and in what procedural environment [19]. In the spirit of the aforementioned expert statement, our study reports the first Greek experience with TBCB, having adapted the technique for the diagnosis of DPLDs after proper training in specialized centers, and presents its diagnostic yield and safety data.

## Methods

### Study design and patient selection

This is a retrospective study of 50 patients with indeterminate DPLD (inconsistent with UIP) initially discussed in an MDT setting in either our Hospital of referred from MDTs of other Institutions. In all cases, clinical history and laboratory/radiological findings were not sufficient to reach a diagnosis, and patients were referred for tissues sampling between January 2016 and August 2018. Patient medical records were analyzed and demographic data, high-resolution computed tomographic (HRCT) scans, procedure details and complications, diagnostic results, and pathology were recorded. All patients underwent lung function testing and transthoracic echocardiography whenever deemed necessary by the treating physician.

Patients with poor performance status, significant hypoxemia (PaO2<55 mmHg while receiving 2 L/min of nasal oxygen), forced vital capacity (FVC) <50% predicted, diffusing capacity of the lungs for carbon monoxide (DLCO) <40% predicted and pulmonary arterial systolic pressure (PASP) >40 mmHg were excluded from undergoing bronchoscopy for TBCB. No strict age limit was set, as long as patients met all previous criteria. The appropriate institutional board that approved this retrospective study is the Athens Chest Hospital "Sotiria" Ethics Committee. Informed written consent was obtained from all participants for TBCB procedure. The Ethics Committee of the Institution waived the requirement for a second informed consent regarding the retrospective analysis of the data. The data were fully anonymized during the analysis, and no patient specific data was included in the manuscript, tables or figures.

### Bronchoscopy and cryobiopsy procedure

TBCBs were introduced at our Department in January 2016 and were performed by two interventional pulmonologists (EZ, KS) using a strict protocol. Both pulmonologists were previously trained in centers with large experience in TBCB (Azienda Ospedaliero-Universitaria “Ospedali Riuniti”, Ancona, Italy and Asklepios Fachkliniken, Gauting, Germany, respectively). All procedures were performed in a dedicated bronchoscopy operating room, in an outpatient setting with intubation under moderate to deep sedation and spontaneous breathing under the supervision of the two attending interventional pulmonologists and two registered nurses.

Anticoagulants were discontinued before the procedure as per established guidelines [20], with the exception of salicylic acid (allowed until 48 hours prior to intervention). Topical anesthesia of the oropharynx, vocal cords, and upper trachea with lidocaine was initially performed. Prior to intubation, a deflated 7 or 9 French Arndt bronchial blocker (Cook Medical, Bloomington, Indiana) was routinely placed through the nose external to the tube and guided with a loop via the bronchoscope towards the segment to be biopsied, to occlude the airway in case of bleeding [21]. When the bronchial blocker was in place at the opening of the targeted segment, the loop was loosened, the blocker was inflated and re-tested for leakage and proper positioning. The blocker was then fixed onto the nose of the patient. In case bronchoalveolar lavage (BAL) was also required, it was performed on the same side as the targeted lobe prior to intubation according to established guidelines [22].

Patients were thereafter fiberoptically intubated with an 8.0 or 8.5-cuffed endotracheal tube, under moderate to deep sedation and spontaneous breathing in the bronchoscopy unit with the bronchial blocker remaining in place external to the tube. Oxygen supplementation was administered through a T-piece swivel adapter. The bronchoscope (BF-1T180 Video Bronchoscope, Olympus Medical, Hamburg, Germany) was advanced to the targeted segment and a 1.9 mm cryoprobe (Erbe Elektromedizin GmBH, Tubingen, Germany) connected to a cryotherapy unit (ERBECRYO 2, Erbe, Germany) was introduced through the working channel of the bronchoscope and moved forward distally until it could not advance further. Prior to obtaining the biopsy, we always tried inserting the probe in at least three subsegmental bronchi of the radiologically most affected segment (areas with ground-glass opacities were preferred), to assess the optimal length of insertion and select the best subsegment for sampling. Most often TBCBs were taken from the lower lobes, which is somewhat expected in diffuse interstitial lung diseases as in most cases the lower lobes have extensive findings in the corresponding CT scans. We then gently retracted the cryoprobe by approximately 1–2 cm, cooled it for 3–5 sec and firmly pulled back separating the frozen biopsy sample from the lung. The sample was extracted along with the bronchoscope and thawed in saline at room temperature.

Upon extraction of the bronchoscope, the blocker was immediately inflated prophylactically to prevent any potential bleeding while the specimen thawed and separated from the tip of the probe. The blocker remained blocked until re-insertion of the bronchoscope and then slowly deflated to check for bleeding. Only one side was biopsied per patient and up to 5 cryobiopsy samples were obtained sequentially from one or two segments of the same lobe. Chest radiography was performed within one hour after the end of the procedure to confirm that a pneumothorax had not taken place. Patients were monitored for at least 4 hours and were discharged the same day after verifying that there were no immediate complications. Follow-up was conducted on all patients at 24–48 hours via telephone to check for possible delayed complications.

### Tissue samples and processing

Cryobiopsies were transferred from saline to 10% formalin within 10 min. Subsequently, they were embedded in paraffin and stained with hematoxylin and eosin, with additional stains at the discretion of 1 of 2 expert lung pathologists. Tissue samples were considered adequate if they exceeded a minimum diameter of 5mm.

### Definition and management of complications

Bleeding severity was defined using a 4-grade system depending on the amount of bleeding after the deflation of the blocker and classified as follows:

- Grade 1, no bleeding
- Grade 2, mild bleeding requiring gentle suction through the bronchoscope
- Grade 3, moderate bleeding requiring intrabronchial epinephrine or cold saline instillation and/or blocker re-inflation for at least 3 min and
- Grade 4, severe bleeding causing hemodynamic or respiratory instability and requiring tamponade and/or other surgical interventions and/or transfusion.

Pneumothorax severity was classified as either mild or severe as follows:

- Mild pneumothorax, less than 2 cm rim present between the lung edge and chest wall, requiring only high flow oxygen supplementation and
- Severe pneumothorax, more than 2 cm rim present between the lung edge and chest wall, requiring chest drain insertion.

### Statistical analysis

Descriptive statistics were used to analyze patient characteristics. Data were checked for normal distribution and described as means and standard deviations (SD). The categorical variables were reported as number of subjects per total or in percentages of total subjects. A statistical software package was used for all data analysis and graph preparation (Prism v6; GraphPad, San Diego, CA, USA).

## Results

### Subjects, procedure and cryobiopsy characteristics

A total of 50 patients (21 F/29 M) with DPLD referred to our Department for TBCB between January 2016 and August 2018. The median age was 61 years (range 43–81), with 60% being current or ex-smokers. Pulmonary function tests were moderately reduced (mean FEV_1_ = 65% pred, mean FVC = 70% pred), while oxygenation was fairly well preserved (mean DLCO = 63% pred and SpO_2_ = 94%). Pulmonary artery systolic pressure (PASP) was evaluated in a proportion of patients (n = 24) and was slightly increased in some subjects (mean PASP = 25 mmHg). Demographic characteristics of the patient population are presented in Table 1.

**Table 1:** Patient characteristics.

Number of patients (N)	50
Gender (F/M)	21 (42%) / 29 (58%)
Age	61 ± 9.8 (43–81)
Smoking status (never/former/current)	20 (40%) / 19 (38%) / 11 (22%)
BMI	29.6 ± 2.9 (23.3–36)
SpO_2_	94.3 ± 14 (92–98)
FEV_1_ predicted %	65.1 ± 7.7 (48–81)
FVC predicted %	69.7 ± 7.9 (52–85)
DLCO predicted %	63.1 ± 8.1 (47–78)
PASP mm Hg *	24.6 ± 5.1 (18–34)

Results presented as mean ± SD with range in parenthesis or N cases with percentage in parenthesis, according to data format (numeric or descriptive respectively).

* Assessed in 24 patients with Echo Doppler ultrasonography

A mean number of approximately 3 TBLCs were taken per patient (with a range from 1 to maximum 5). The mean diameter of the cryobiopsies was 5.1 mm ± 1.8 mm, in the range of size suggested as sufficient for histopathological diagnosis in the recent expert statement [19]. Most often (41 patients; 82%), TBLBs were taken from the lower lobes (right side: 25 (50%), left side: 16 (32%). In 60% of the cases, TBLBs were obtained from one bronchial segment, while in the remaining 40% two segments were biopsied, but always in the same lobe. Biopsy samples did not contain pleural tissue or crush artifacts. Procedure and biopsy characteristics are presented in Table 2.

**Table 2:** Procedure and biopsy characteristics.

*Cryobiopsies*	
No of biopsies taken per patient	2.75 ± 0.9 (1–5)
Mean size in mm	5.16 ± 1. 83 (2–16)
*Location*	
Left Upper Lobe	1 (2%)
Left Lower Lobe	16 (32%)
Right Upper Lobe	1 (2%)
Middle Lobe	7 (14%)
Right Lower Lobe	25 (50%)
One segment	30 (60%)
Two segments	20 (40%)

Results presented as mean ± SD with range in parenthesis or N cases with percentage in parenthesis, according to data format (numeric or descriptive respectively)

### Major complications and safety

The complications of the procedure are reported in Table 3. Bleeding as a complication was frequently observed in the majority of our patients (62%) but, in most cases (38%), it was rather mild requiring only gentle suction. Moderate bleeding was observed in 12 patients (24%) and was easily treated by instilling cold saline or in some cases by re-inflating the blocker for at least 3 minutes. Severe bleeding resulting in hemodynamic instability or requiring transfusion was not observed in any of our patients.

**Table 3:** Complications.

Bleeding	
None	19 (38%)
Mild	19 (38%)
Moderate	12 (24%)
Severe	0
Pneumothorax	
Mild	5 (10%)
Severe	0

Bleeding severity scale: no bleeding, mild bleeding (requiring suction to clear but no other endoscopic procedures), moderate bleeding (requiring instillation of ice-cold saline and/or adrenaline and/or bronchial blocker re-inflation), and severe bleeding (causing hemodynamic or respiratory instability, requiring tamponade, transfusions or admission to the intensive care unit). Pneumothorax severity scale: mild (small pneumothorax >2 cm rim present between the lung edge and chest wall, requiring only high flow oxygen supplementation), severe (large pneumothorax <2 cm rim present between the lung edge and chest wall, requiring active intervention (requiring chest drain insertion).

Pneumothorax was observed in 5 patients (10%) and all cases were mild. In three of the cases the pneumothorax was identified immediately after the TBLC procedure when patients were checked with chest radiography. The other two patients were discharged from the hospital with no clinical or imaging findings of pneumothorax, but presented to our department the next day with pleuritic chest pain starting approximately 12 hours after the TBLC procedure. A chest X-ray confirmed the presence of pneumothorax and both patients were readmitted. In all cases pneumothorax was mild (<2 cm rim present between the lung edge and chest wall), and patients were treated with high flow oxygen supplementation, requiring hospitalization for less than 3 days.

### Diagnosis

The most frequent HRCT pattern observed in patients at the time of inclusion was ground glass opacities (50%) and, usually bilateral, consolidations (40%), followed by reticular (30%) and reticulonodular findings (18%). Traction bronchiectasis/bronchiolectasis was observed also in a substantial part of the patients, while sparce honeycombing was found in a small minority of patients. HRCT findings for all patients are represented in Table 4.

**Table 4:** HRCT findings at time of inclusion.

HRCT findings observed	No of patients	% of patients
Consolidation	20	40%
Ground glass opacities (GGO)	25	50%
Emphysema	5	10%
Bronchiectasis	14	28%
Reticular pattern	15	30%
Nodular pattern	10	20%
Reticulonodular pattern	9	18%
Cystic lesions and/or honeycombing	9	18%
Mediastinal lymph node enlargement	2	4%
Pleural effusion/thickening	1	2%

Adequate samples for diagnostic purposes were obtained in 46 patients (92%) and histologic diagnosis was reached in 40 patients (80%). The most frequent histopathological patterns were organizing pneumonia (25%) and non-specific interstitial pneumonia/NSIP (15%). Histopathological diagnosis of all cases is presented in Table 5.

**Table 5:** Histopathological diagnosis based on cryobiopsies.

Histological diagnosis	No of patients	% of patients
Adequate samples	46	92%
UIP	3	6%
NSIP	8	16%
Compatible with DIP	1	2%
RB	3	6%
OP	13	26%
HP—consistent	4	8%
Malignancy	1	2%
Lymphoid hyperplasia*	3	6%
Granulomatous inflammation	3	6%
EP	2	4%
Normal tissue	2	4%
No diagnostic pattern	3	6%
Inadequate samples (<5mm)	4	8%

NSIP: non-specific interstitial pneumonia; UIP: usual interstitial pneumonia; HP: hypersensitivity pneumonia; DIP: desquamative interstitial pneumonia; EP: eosinophilic pneumonia; OP: organizing pneumonia; RB: respiratory bronchiolitis.

* Two cases with follicular bronchiolitis and one case with lymphoid interstitial pneumonia (LIP).

**Fig 1:**
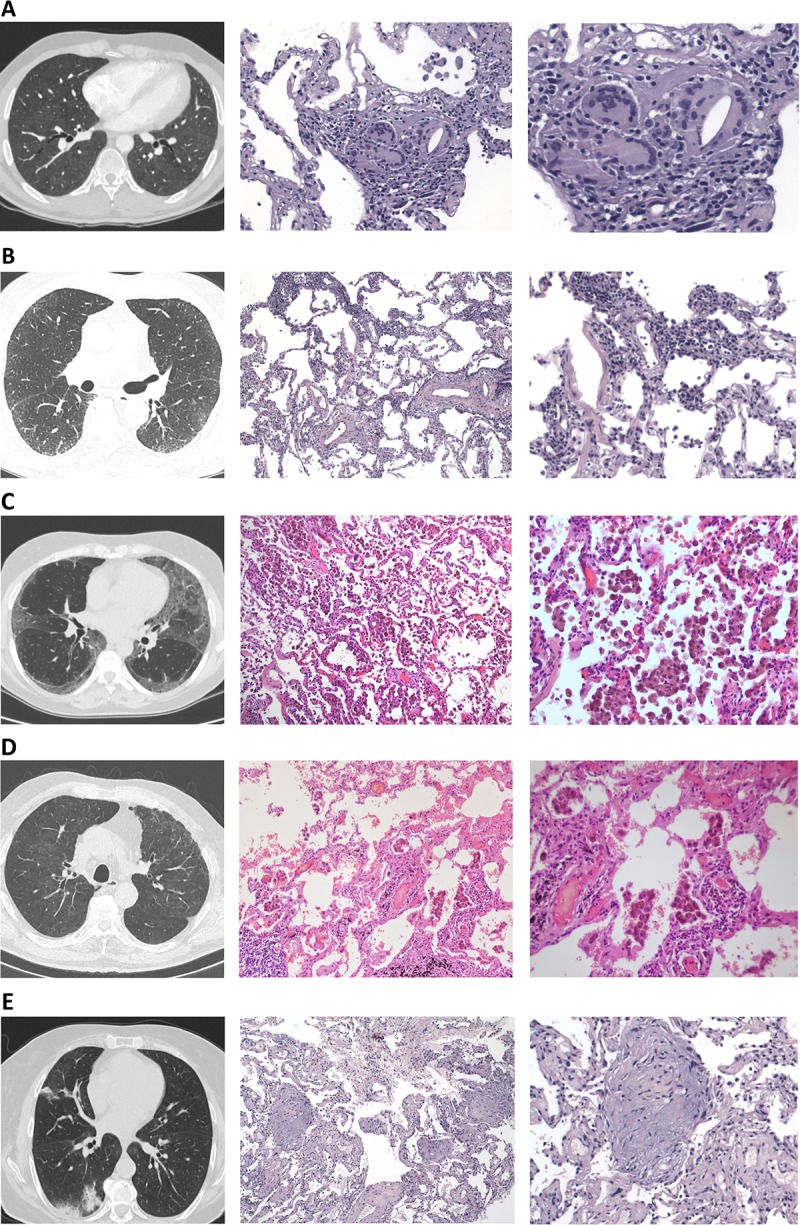
HRCT patterns and histology findings. (A) Patient 1: HRCT (pulmonary window) demonstrating diffuse ground glass opacities, small ill-defined centrilobular nodules, lobular areas of decreased attenuation (mosaic pattern) with a histological pattern consistent with hypersensitivity pneumonitis demonstrating poorly formed non-necrotizing granulomas (x20 and 40 respectively) and a definite diagnosis after MDT review of hypersensitivity pneumonitis. (B) Patient 2: HRCT (pulmonary window) demonstrating areas of ground glass opacities mainly involving the left lower lobe and mild peripheral reticulation with a histological pattern of cellular non-specific interstitial pneumonia (NSIP) with diffuse, evenly distributed, moderate chronic inflammation, without effacement of the alveolar architecture or evident granulomas a patient diagnosed with (x10 and 20 respectively) and a definite diagnosis after MDT review of undifferentiated form of CT-ILD. (C) Patient 3: HRCT (pulmonary window) demonstrating bilateral areas of ground glass opacities with peripheral and subpleural predominance with a histological pattern compatible with desquamative interstitial pneumonia (DIP-like reaction) with intraalveolar macrophage aggregates and alveolar septae with mild focal interstitial fibrosis (x10 and 20 respectively) and a definite diagnosis after MDT review of smoking related interstitial lung disease (SR-ILD). (D) Patient 4: HRCT (pulmonary window) demonstrating bilateral patchy areas of ground glass opacity, mild subpleural intralobular linear opacities with a histological pattern of respiratory bronchiolitis (RB) with the presence of histiocytes in a centroacinar rather than in a diffuse pattern (x10 and 20 respectively) and a definite diagnosis after MDT review of smoking related interstitial lung disease (SR-ILD). (E) Patient 5: HRCT (pulmonary window) demonstrating sub-pleural and peribronchial consolidation in the posterior segment of right lower lobe and middle lobe with a histological pattern of organizing pneumonia (OP) showing the presence of exudate of inflammatory cells, foam cells, fibroblastic Masson bodies and macrophages creating a bronchiolar obstruction (x10 and 20 respectively) and a definite diagnosis after MDT review of cryptogenic organizing pneumonia (COP).

Final diagnoses after expert panel review of the cases are presented in Table 6. Cryobiopsies contributed to a final diagnosis in 38 patients, corresponding to a diagnostic yield of 76%. The most frequent diagnoses reached were COP (16%) and HP (14%). SLB was suggested in five patients but only two accepted to undergo the procedure; one was diagnosed with B-cell low-grade lymphoma and the other with fibrotic NSIP. In other patients without definite diagnosis, observation without further invasive investigation was recommended, and regular follow-up visits were scheduled. Examples of representative HRCT findings and histology pictures are depicted in Fig 1.

**Table 6:** Final clinical diagnosis after expert panel review.

Final clinical diagnosis	No of patients	% of patients
IPF	3	6%
NSIP	1	2%
CTD-ILD	3	6%
Undifferentiated forms of CTD-ILD	3	6%
SR-ILD	4	8%
DR-ILD	2	4%
COP	8	16%
HP	7	14%
EP (chronic)	1	2%
Lymphoma	1	2%
Sarcoidosis	4	8%
Malignancy	1	2%
No diagnosis	12	24%

NSIP: non-specific interstitial pneumonia; IPF: idiopathic pulmonary fibrosis; HP: hypersensitivity pneumonia; CTD-ILD: connective tissue related interstitial lung disease; SR-ILD: smoking related interstitial lung disease; DR-ILD: drug related interstitial lung disease; EP: eosinophilic pneumonia; COP: cryptogenic organizing pneumonia.

## Discussion

This study presents the first Greek experience with the introduction of TBCB technique in the diagnosis of DPLDs. All procedures were performed by two interventional pulmonologists with prior training on TBCB in experienced centers, and both physicians were present during each bronchoscopy. In this series of 50 patients with “inconsistent with UIP” DPLDs, adequate lung tissue was obtained in 46 cases and when combined with clinical and radiological information a definite diagnosis after expert panel review was reached in 76% of the cases. This is considerably higher than landmark studies evaluating the use of traditional forceps transbronchial biopsies [23–25]. Moreover, our diagnostic yield is somewhat lower than that of other experienced groups [9,13,26–29], it is however comparable to the diagnostic yield of groups with less experience that have just introduced TBCB to their centers [30–34], and this shows that TBCB is a procedure worth investing in.

Previous studies have shown that sample number, size and site of origin may directly influence the diagnostic yield of TBCB. The mean diameter of the cryobiopsies in our study was approximately 5 mm, which is over the threshold suggested as sufficient for histopathological diagnosis by expert pathologists [10,11] and the recent TBCB task force [19]. With respect to the number of biopsies, we aimed to obtain at least three samples per patient, which was accomplished in most cases. In three cases we obtained only one biopsy due to moderate bleeding after the extraction followed by patient discomfort, and these samples were considered inadequate during analysis. Regarding the site of origin, most often TBCBs were taken from the lower lobes, which is somewhat expected in diffuse interstitial lung diseases as in most cases the lower lobes have extensive findings in the corresponding CT scans. For safety reasons, we performed TBCB in only one lobe, albeit in different segmental bronchi in 40% of the cases, as this has recently been shown to increase the diagnostic yield without raising complication rates [29]. Sampling from two different lobes may increase further the diagnostic yield of the procedure especially when different patterns are presented in different lobes in HRCT. Nevertheless, the only published study where multiple lobes were involved (64% of patients) reported an very high complication rate [35], which was however not directly associated with the site of sampling. Sampling from different lobes is not suggested in the recently published expert statement on TBCB [19], further studies are needed to clarify associated safety issues.

The most frequent final diagnoses obtained were COP (16%) and HP (14%), followed by IPF, SR-ILD and sarcoidosis. Reported rates for organizing pneumonia and HP are rather high in our cohort. The prevalence of HP may vary considerably in different countries and even within the same country due to geographic, seasonal, and climatic factors [36]. Furthermore, COP prevalence in Greece has previously been reported to be high in Greece, representing more than 60% of all biopsy proven OP patients [37]. In any case, COP and HP rates reported in our study are comparable to previously published case series on cryobiopsies from Belgium, Denmark and Portugal [30,32,33] and corresponding European registries for ILDs [38]. In our study, OP was a histological (not a definite) diagnosis reached in 13 cases, however after careful consideration in an MDT setting in 5 cases an alternate definite diagnosis other than COP was reached based on clinical/laboratory data, whereas in 8 cases COP was the final clinical diagnosis after excluding all other possibilities.

In our study a total of six patients had features of CT-ILD; however only three had an underlying connective tissue disease (CTD) with positive serum markers and relevant clinical symptoms leading to a definite diagnosis of CT-ILD. The remaining three cases did not meet the criteria for CTD but demonstrated corresponding clinical, serological or morphological features or similar disease behavior and were therefore diagnosed with undifferentiated forms of CTD-ILD. An official ERS/ATS statement has recently set specific criteria to define this population as Interstitial Pneumonia with Autoimmune Features (IPAF); however this term was conceptualized for future research framework [39].

Regarding the pathologic diagnoses of RB and DIP in three cases, after careful consideration of all clinical, laboratory and HR-CT data in the expert review panel, another cause could not be identified (such as evidence of a CTD disorder, inhalational injuries, drug or other agent exposure etc., that could be strictly related with the above mentioned pathological diagnoses). Therefore, as these patients were heavy smokers, the MDT considered that these cases were smoking related RB-ILD and DIP respectively, and were all labelled as SR-ILD, as they share a number of clinical, radiologic and pathologic features suggesting that they represent a spectrum of patterns of interstitial lung disease occurring in predisposed individuals who smoke. Moreover, the rate of UIP histology pattern and IPF final diagnosis was relatively lower in our cohort compared to previously reported studies. This can be attributed to bias as patient enrollment was not consecutive, as some patients with inconsistent with UIP patterns in HRCT were excluded in the light of recent diagnostic criteria underlying the importance of HRCT together with MDT evaluation to reach a confident working diagnosis of IPF [1,40,41].

Complications namely bleeding and pneumothorax were rather low in our study and comparable with the rates reported in the literature [42]. Reported pneumothorax rates vary substantially between studies from 0% up to over 30% [42]. In two recent meta-analyses that included 13 and 15 studies respectively the overall incidence of pneumothorax was approximately 10% [12,13]. Our relative low rates and severity of pneumothorax may be attributed to the small number of patients with UIP histology and fibrotic changes in HRCT, and the lack of pleural tissue in biopsy samples, factors associated with high risk for pneumothorax [13,26]. Three out of five pneumothoraces were observed after obtaining TBCBs in the middle lobe. Given that pneumothorax in the middle lobe occurred in three out of seven patients suggests that the middle lobe and possibly the lingula should probably be avoided as targets for TBCB. Furthermore, two pneumothorax cases developed a day after the biopsy, suggesting that mechanisms other than direct trauma or mechanical disruption might contribute to its pathogenesis. It seems therefore important to follow up patients closely for a few days after the procedure. In our study, bleeding rates and severity were also within reported ranges in our study, with only mild and moderate cases observed. As the expert statement suggested, using a blocker is essential in greatly reducing the incidence of severe bleeding. Proper training prior to introducing the technique and the fact that we used a thin 1.9 mm cryoprobe might have also contributed to this outcome. No other complications such as infections or acute exacerbations occurred in our study.

Despite the promising diagnostic yields of TBCB reported in the literature, most ILD experts worldwide are still reluctant to adopt this new technique in their diagnostic algorithm. This is mainly due to the lack of data on the direct comparison of TBCB and SLB in the same patient population with specific HRCT findings (inconsistent vs consistent UIP), the lack of standardization until now with respect to both patient selection and the procedure, as well as several safety issues raised especially in the US [35]. The recently published expert statement represents an important step forward in standardizing TBCB with respect to the necessary equipment, indications and contraindications, risks and training requirements containing several key-points after each section that are meant as expert suggestions to interventional clinicians, therefore facilitating uniform practice and guidance for those wishing to introduce this technique in their departments [19]. The existence of these expert recommendations along with the promising data accumulated during the last few years, may reassure medical communities and health systems to endorse a wider adoption of this technique.
